# Immature Granulocyte Trajectories Following Hemadsorption as Indicators of Immune Dysregulation and Mortality

**DOI:** 10.3390/jcm15031011

**Published:** 2026-01-27

**Authors:** Gülsüm Altuntaş, Ayşe Çapar, Gülsüm Özçelik, Erkan Çakmak, Lütfiye Kadioğlu Dalkiliç, İsmail Demirel

**Affiliations:** 1 Department of Anesthesiology and Reanimation, Faculty of Medicine, Firat University, Elazig 23300, Turkey; gulsumozcelik2012@gmail.com (G.Ö.); ismaildemirel23@firat.edu.tr (İ.D.); 2 Anesthesiology and Intensive Care Medicine, Istanbul Sultan Abdulhamid Han Training and Research Hospital, Health Sciences University, Istanbul 34668, Turkey; drayseyel@hotmail.com; 3 Department of Internal Medicine, Faculty of Medicine, Firat University, Elazig 23300, Turkey; drerkan_23@hotmail.com; 4 Department of Molecular Biology and Genetics, Faculty of Science, Firat University, Elazig 23119, Turkey; tkadioglu85@gmail.com

**Keywords:** sepsis, Hemoperfusion, Granulocytes, Immature, intensive care

## Abstract

**Background:** Sepsis is a life-threatening condition characterized by a dysregulated host response to infection. Hemadsorption therapies remove inflammatory mediators and are used as adjunctive treatment in selected patients. Although increased immature granulocyte (IG) levels correlate with inflammatory severity, changes in IG levels after hemadsorption therapy have not been previously evaluated. **Methods:** This retrospective observational study included patients with sepsis who received hemadsorption therapy in intensive care units between January 2021 and July 2025. Sepsis was diagnosed according to the Surviving Sepsis Campaign 2021 guidelines, and hemadsorption was initiated for persistent hemodynamic instability despite standard therapy. Treatment was performed using a Jafron HA330 cartridge for at least three 6 h sessions. IG count and percentage, inflammatory parameters, lactate levels, and organ dysfunction scores were recorded before and after therapy. ICU mortality was the primary outcome. Statistical analyses included paired comparisons, multivariable logistic regression, and ROC curve analysis. **Results:** Among 887 patients with sepsis, 196 met the inclusion criteria. The ICU mortality rate was 43.9%, and the median time between pre- and post-treatment measurements was 4 days (IQR: 3–5). After hemadsorption therapy, IG count, IG%, inflammatory parameters, lactate levels, SOFA scores, and vasopressor requirements decreased (all *p*-values < 0.001). IG parameters were higher in non-survivors. Post-treatment IG# (AUC 0.880) and IG% (AUC 0.812) showed good discriminative performance. **Conclusions:** Hemadsorption therapy was associated with reductions in IG parameters and inflammatory indicators in sepsis. These findings support IG parameters as complementary measures of immune and inflammatory dynamics during hemadsorption therapy. Accordingly, this study should be regarded as a hypothesis-generating investigation describing associations of IG dynamics in septic patients undergoing hemadsorption, rather than demonstrating treatment efficacy or causal effects.

## 1. Introduction

Sepsis is a leading cause of morbidity and mortality among intensive care patients. It is defined as life-threatening organ dysfunction caused by a dysregulated host response to infection [1]. Immune activation leads to the release of proinflammatory cytokines. Procalcitonin (PCT), C-reactive protein (CRP), interleukin-6 (IL-6), and tumor necrosis factor α (TNF) are commonly elevated in sepsis [2]. However, obtaining these laboratory measurements may be limited by cost and availability in routine clinical practice [3].

Additionally, during inflammatory conditions such as sepsis, there is bone marrow hyperactivation and a tenfold increase in neutrophil production, known as a leukemoid reaction. The immature neutrophils produced are released into the peripheral circulation [4]. These neutrophil precursors, called immature granulocytes (IG), include promyelocytes, myelocytes, and metamyelocytes [5]. They can be easily measured as numbers and percentages using automated hematology analyzers. Unlike conventional inflammatory parameters such as CRP or procalcitonin, which primarily reflect downstream cytokine-mediated responses, IG represents a cellular component of emergency granulopoiesis and bone marrow activation during systemic inflammation [4]. Their presence in peripheral blood reflects dysregulated innate immune responses and impaired neutrophil maturation, the pathophysiological features of sepsis [6,7]. Several clinical studies have demonstrated that IG parameters correlate with sepsis severity and may provide prognostic information complementary to established biomarkers and clinical severity scores [8,9,10,11,12].

Laboratory parameters evaluated in this context are not intended as decision-making tools, but rather as complementary measures to describe immune and inflammatory dynamics during critical illness.

Treatment for sepsis and septic shock includes fluids, vasopressors, appropriate antibiotic therapy, focus control, mechanical ventilation support, and renal replacement therapy [13]. However, new and complementary treatment approaches have been developed to help reduce the dysregulated inflammatory response in sepsis. One of these treatments is extracorporeal cytokine hemadsorption. These therapies aim to reduce circulating pathogens and inflammatory mediators, including cytokines, through extracorporeal removal from the blood [14].

One of the leading selective hemadsorption systems in this field is the Jafron HA330 cartridge. The device features a porous resin structure with a large surface area (300–1200 m^2^/g) that can remove cytokines, complement proteins, and various endotoxins in the 10–60 kDa range [15]. As blood passes through the cartridge containing adsorbent microbeads, the target molecules are captured and eliminated by the porous structure. Adsorption occurs through hydrophobic interactions, van der Waals forces, and ionic bonds [14]. In addition to managing the hyperinflammatory response, it may also improve organ function and hemostasis [16].

However, studies have shown that increased IG is associated with the severity of inflammation [7,8,9,10,11,12,17,18], yet no research has examined changes after hemadsorption. Accordingly, this study retrospectively analyzes changes in laboratory parameters and clinical outcomes in septic patients undergoing hemadsorption. It aims to explore the associations and temporal patterns of IG parameters during the ICU course rather than assessing treatment effects or inferring causality.

## 2. Materials and Methodology

### 2.1. Study Design and Patients

This retrospective observational study included patients 18 years of age or older who were diagnosed with sepsis and received hemadsorption therapy in our tertiary intensive care units. The study included patients who received hemadsorption therapy, with within-patient pre- and post-treatment comparisons performed to descriptively assess temporal changes in laboratory parameters. The Fırat University Non-Interventional Research Ethics Committee approved the study on 11 September 2025 under number 38820. Our study adhered to the principles of the Helsinki Declaration and followed the Strengthening the Reporting of Observational Studies in Epidemiology (STROBE) guidelines.

Patients under 18 years old, those discharged, transferred, or deceased within 72 h of a sepsis diagnosis, pregnant women, patients with missing data, patients who did not receive hemadsorption therapy, patients diagnosed with COVID-19, and patients with a history of malignancy, hematological disease, or immunosuppression were excluded from the study. Because of the study’s retrospective design, informed consent was waived.

### 2.2. Data Collection

Data from January 2021 to July 2025 were collected from patient records and the hospital’s electronic database. Demographic information, including age, gender, Charlson Comorbidity Index, Glasgow Coma Scale (GCS), Acute Physiology and Chronic Health Evaluation II (APACHE II) score, and Sequential Organ Failure Assessment (SOFA) score, was retrieved from the hospital database. The need for mechanical ventilation (MV), renal replacement therapy (RRT), and vasopressor dosages was obtained from patient files.

Sepsis was diagnosed by experienced intensive care specialists in accordance with the Surviving Sepsis Campaign 2021 guidelines [13]. All patients received guideline-based standard sepsis management, including early initiation of broad-spectrum antimicrobial therapy with subsequent adjustment according to microbiological findings and clinical response. Source control interventions, such as drainage, surgical procedures, or removal of infected devices, were performed when indicated. Initial hemodynamic management consisted of intravenous fluid resuscitation, followed by vasopressor support in patients with persistent hypotension, targeting recommended mean arterial pressure goals. Vasopressor selection and dose titration were guided by continuous hemodynamic monitoring and individual patient response. Additional supportive therapies, including mechanical ventilation and renal replacement therapy, were applied as clinically required. Decisions regarding antibiotic timing, source control strategies, fluid resuscitation, and vasopressor titration were made by attending intensivists based on illness severity and the dynamic clinical course. Due to the retrospective design and heterogeneity of clinical management, these treatment-related variables were not analyzed quantitatively. Hemadsorption therapy was administered at the discretion of the intensive care specialist, based on the literature [16], in cases of hemodynamic instability despite conventional treatment (fluids, vasopressors, antibiotics). The durations of mortality and ICU stay (LOS) were recorded. Mortality was defined as death occurring during the ICU stay.

Laboratory parameters, including complete blood count (Hemoglobin [Hb], Hematocrit [Hct], White Blood Cell [WBC], Neutrophils, Lymphocytes, IG#, IG%, Platelets, Monocytes), along with Urea, Creatinine, Albumin, CRP, Procalcitonin, D-Dimer, INR, SOFA score, and vasopressor dose requirement, were recorded on the day the hemadsorption treatment began and ended.

Hemadsorption therapy was performed using a 12F hemodialysis catheter placed in the internal jugular or femoral vein and connected to a Jafron HA 330 filter (HA 330, Jafron Biomedical Co., Ltd., Zhuhai, China). Hemadsorption therapy was administered in at least three sessions, each lasting 6 h, according to the institutional protocol. Pre-treatment laboratory samples were obtained on the day hemadsorption therapy was initiated, and post-treatment samples were collected after completion of the final hemadsorption session. The time interval between these two sampling points was calculated for each patient and summarized using the median and interquartile range. When combined with continuous renal replacement therapy (CRRT), the blood flow rate was 100 mL/min, and citrate was used for anticoagulation. If administered separately, the blood flow rate ranged from 160 to 180 mL/h, with anticoagulation using 5000 units of unfractionated heparin during priming. Complete blood count, Ig count, and percentage were measured using the Sysmex XE-5000 device (Sysmex, Kobe, Japan).

### 2.3. Outcome Points

Primary: Changes in inflammatory parameters, including CRP, procalcitonin, IG#, IG%, neutrophil and lymphocyte counts, lactate, and albumin, were measured before and after hemadsorption therapy.

Secondary endpoints included changes in APACHE II and SOFA scores, the need for mechanical ventilation and vasopressors, and mortality.

### 2.4. Statistical Analyses

Analyses were conducted using SPSS v30.0 (IBM Corp., Armonk, NY, USA). Descriptive statistics are presented as mean ± standard deviation or median (Q1–Q3), depending on the distribution; categorical variables are expressed as n (%). Normality was assessed with the Kolmogorov–Smirnov test. For group comparisons, the independent-samples *t*-test or Mann–Whitney U test was used based on the distribution of the variables, and the chi-square test was applied for categorical variables. The Wilcoxon Signed-Rank test was used to compare pre- and post-treatment values. To identify factors associated with mortality, an initial univariate logistic regression analysis was performed, followed by inclusion of significant variables in the multivariate model. Multivariate logistic regression was conducted using the 8-step Backward Wald method. The model’s fit was evaluated using the Hosmer–Lemeshow test (*p* = 0.192), and multicollinearity was assessed using VIF < 5 and tolerance > 0.8.

To assess the discriminatory ability of the variables for mortality, ROC (Receiver Operating Characteristic) curve analysis was conducted. For each parameter, the AUC (Area Under the Curve), 95% confidence interval, cutoff value, sensitivity, specificity, and Youden index were calculated.

## 3. Results

**Figure 1:**
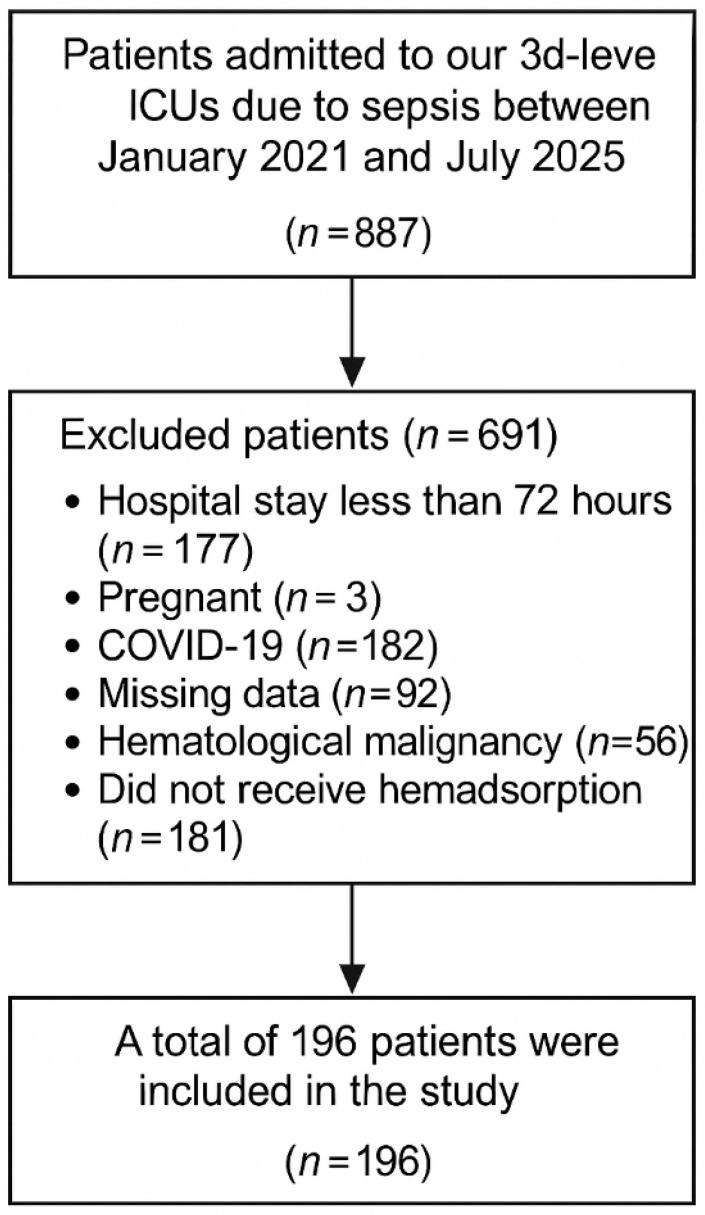
Flow Diagram of Patients.

Between January 2021 and July 2025, 887 patients were admitted to our third-level ICUs with sepsis. We excluded 177 patients with hospital stays less than 72 h, 3 patients due to pregnancy, 182 patients with COVID-19, 92 patients due to missing data, 56 patients with hematological malignancy, and 181 patients who did not undergo hemadsorption. A total of 196 patients were included in the study (Figure 1).

Among patients, 58.7% were male (*n* = 115) and 41.3% were female (*n* = 81), with an average age of 57.0 ± 14.7 years. The average Charlson Comorbidity Index was 2.0 (1.0–2.0). The median length of stay in the intensive care unit was 12 (8–17) days, and the duration of mechanical ventilation was 8 (5–12) days. Eighty-two point seven percent of patients received mechanical ventilation, fifty-seven point seven percent received RRT, and the mortality rate was 43.9%. The APACHE II score averaged 21.8 ± 3.4, and the pre-treatment GCS score was 12 (9–14). The vasopressor dose (VP) before treatment was 0.58 (0.35–0.85) µg/kg/min, while after treatment it was 0.09 (0.02–0.40) µg/kg/min. The median time between pre- and post-treatment laboratory sampling was 4 days (interquartile range: 3–5 days) (Table 1).

The median SOFA score before treatment was 11 (range 8–12), while it was 8 (range 3–11) after treatment (*p* < 0.001). The IG count decreased from 0.45 (0.27–0.77 × 10^3^/µL) to 0.09 (0.06–0.28), and the IG percentage (IG%) decreased from 2.50 (1.83–3.88) to 0.80 (0.40–1.50) (*p* < 0.001). WBC decreased from 19,650 (15,520–25,910/mm^3^) to 11,610 (8472.5–17,077.5/mm^3^), neutrophils decreased from 16,840 (12,490–22,542.5) to 9320 (6087–14,987.5) (*p* < 0.001). Lymphocytes increased from 720 (475–957.5) to 1100 (860–1502.5) (*p* < 0.001), monocytes decreased from 980 (680–1210) to 560 (340–808.5) (*p* < 0.001). There was no difference in platelet levels (185,000→184,000, *p* = 0.216). Lactate decreased from 11.15 (8.12–13.10) to 2.10 (1.33–3.80), CRP from 183 (135–219) to 72.85 (29.78–111.00), PCT from 11.14 (3.60–26.53) to 1.90 (0.71–4.50) (all *p* < 0.001). Albumin decreased from 2.90 (2.60–3.40) to 2.80 (2.50–3.10) (*p* = 0.021), and D-dimer decreased from 3.90 to 2.89, but the differences were not significant (*p* = 0.073). Vasopressor dose decreased from 0.70 (0.40–1.00) to 0.10 (0.03–0.50 µg/kg/min) (*p* < 0.001) (Table 2).

The mortality rate was 43.9% (*n* = 86). Patients who died had a higher Charlson comorbidity score [2.5 (2.0–3.0) vs. 2.0 (1.0–2.0); *p* < 0.001], a lower GCS [10 (8–12) vs. 12 (8–14); *p* < 0.001], and a higher baseline SOFA score [12 (10.75–13.00) vs. 8 (6–11); *p* < 0.001]. SOFA scores remained elevated in the mortality group after hemadsorption therapy [10 (9–12) vs. 3 (2–4.25); *p* < 0.001]. Additionally, APACHE II scores were significantly higher in the mortality group [28.0 ± 4.76 vs. 18.4 ± 5.86; *p* < 0.001]. The pre-treatment IG value was 0.67 (0.45–0.89) among those who died and 0.32 (0.21–0.44) among those who survived (*p* < 0.001). The post-treatment IG value decreased from 0.22 (0.10–0.38) to 0.06 (0.03–0.07) (*p* < 0.001).

IG% values were also higher in the mortality group (pre-treatment 2.90 vs. 2.15, post-treatment 1.40 vs. 0.50, both *p* < 0.001). The vasopressor dose was higher in those who died (pre-treatment 0.6 vs. 0.7, *p* = 0.007; post-treatment 0.4 vs. 0.02, *p* < 0.001). The need for RRT was 75.6% in the mortality group and 43.6% in the survival group (*p* < 0.001). Similarly, the requirement for mechanical ventilation was higher in the mortality group (95.3% vs. 72.7%; *p* < 0.001) (Table 3). Changes in other laboratory parameters between deceased and surviving patients are shown in Table 3. Exploratory multivariable analyses are provided in the Supplementary Material (Table S1).

In ROC analyses, IG# and IG% parameters demonstrated good discriminatory ability for mortality. Pre-treatment AUC for IG#: 0.768 (95% CI: 0.699–0.836), cut-off: 0.425, sensitivity: 80.2%, specificity: 69.1%, Youden index: 0.493 (*p* < 0.001).

The post-treatment IG# was identified as the parameter with the highest prognostic power (AUC: 0.880; 95% CI: 0.826–0.935), with a cut-off of 0.085, sensitivity of 90.7%, specificity of 82.7%, and Youden index of 0.734 (*p* < 0.001). For pretreatment IG%, the AUC was 0.706 (95% CI: 0.633–0.780), with a cut-off of 2.15, sensitivity of 82.6%, and specificity of 52.7%. For post-treatment IG%, the AUC was 0.812 (95% CI: 0.746–0.878), with a cut-off of 0.85, sensitivity of 70.9%, and specificity of 86.4% (both *p* < 0.001) (Table 4, Supplementary Figure S1).

## 4. Discussion

In this study, inflammatory, immunological, and clinical changes during the ICU course of septic patients treated with hemadsorption were evaluated in a descriptive framework. In addition to conventional inflammatory markers and clinical severity measures, IG parameters were examined as indicators of innate immune dysregulation. Overall, IG parameters exhibited temporal variation during the clinical course and were associated with clinical outcomes. These findings describe associations and immune trajectories rather than treatment efficacy or causal immunomodulatory effects and should be interpreted within the context of comprehensive sepsis management.

Controlling the infection focus, initiating antibiotics early, and providing appropriate fluid resuscitation are the primary treatment strategies for sepsis [13]. If any of these steps are omitted, the potential impact of adjunctive cytokine removal therapies may be reduced. Given the limited clinical evidence, hemadsorption is currently regarded as a complementary therapy, underscoring the importance of appropriate patient selection. The ADQI-30 working group also highlights in their consensus report the importance of clearly defining target patient groups and developing inflammation-based treatment protocols [16].

Meanwhile, several theories have been proposed to describe the potential effects of hemadsorption therapies and to emphasize the importance of cytokine removal. These include the ‘Peak Concentration Theory’, ‘Threshold Immunomodulation Theory’, and ‘Mediator Delivery Theory’ [19]. It has been suggested that blood purification may be associated with changes in inflammatory mediator dynamics, including reductions in circulating cytokine levels and redistribution of mediators between tissue and blood compartments. None of these theories has yet been fully proven. Although the cost of this treatment is high, it has been used in selected clinical settings, particularly in patients with severe inflammatory burden [20,21].

Several studies have reported laboratory and clinical changes associated with hemadsorption therapy in inflammatory conditions such as sepsis. In their multicenter prospective study, Cuhuli et al. reported that hemadsorption therapy lowered endotoxin levels, reduced rates of renal failure and mortality, and was effective in monitoring endotoxin trends [22]. The prospective study by Koç et al. indicated significant decreases in parameters such as TNF-α, IL-6, IL-10, PCT, and CRP, as well as in APACHE II and SOFA scores [23]. Early use in COVID-19 patients has been shown to significantly decrease respiratory failure, improve oxygenation and organ function, and reduce kidney damage and mortality [24]. Starting treatment within the first hours of pediatric and adult sepsis has been reported to improve hemodynamic stability and organ function, leading to lower-than-expected mortality rates [25,26]. Tanaka et al. observed that hemadsorption therapy reduced mortality more effectively in patients with intra-abdominal infections and attributed this to increased endotoxin filtration and the potential control of the infection focus through surgical intervention [27]. However, reported effects vary across studies and are influenced by differences in patient selection, timing of intervention, infection source, and study design.

In the present study, no distinction was made by site of infection, and patients with sepsis and septic shock were analyzed together to reflect real-world clinical heterogeneity. We observed temporal changes in inflammatory markers and clinical severity measures during the ICU course. These observations likely reflect overall clinical and inflammatory trajectories during comprehensive sepsis management, with hemadsorption applied as part of multimodal supportive care.

During sepsis, systemic inflammation is associated with dysregulated innate immune responses, including emergency granulopoiesis and the premature release of immature granulocytes into the circulation [4,6,7,17]. Thus, IG parameters may reflect aspects of immune dysregulation beyond circulating inflammatory mediators. Hemadsorption therapies are applied in patients with pronounced hyperinflammatory states, and observed changes in IG parameters during the ICU course may reflect evolving immune dynamics. However, these findings should not be interpreted as evidence of direct cellular or bone marrow-specific effects but rather as part of broader inflammatory processes during comprehensive sepsis management.

Numerous studies have explored the association between IG parameters and systemic inflammatory conditions, including sepsis [8,9,10,11]. Collectively, these studies suggest that IG measurements may reflect aspects of innate immune activation and emergency granulopoiesis during severe infection. Ayres et al. reported that IG# showed a stronger association with sepsis-related inflammatory status than CRP and IL-6, while lower IG% values were observed in patients without sepsis [12]. Other investigations have demonstrated associations between elevated IG parameters and disease severity, adverse clinical outcomes, or the need for treatment modification across different clinical settings, including burn-related sepsis, post-cardiac surgery sepsis, and bacterial pneumonia in COVID-19 patients [18,28,29,30]. Together, these findings support the complementary role of IG parameters alongside established inflammatory markers in the clinical assessment of sepsis.

Similarly, in our study, IG parameters were associated with mortality, and ROC analyses suggested discriminatory performance of both IG# and IG% across measurement time points. However, IG parameters may provide complementary information on immune dysregulation during the ICU course rather than representing clinically actionable thresholds. Since the underlying biological mechanisms remain unclear, IG trajectories may reflect immune and inflammatory dysregulation in sepsis rather than direct immunomodulatory effects of hemadsorption.

In exploratory analyses, post-treatment IG parameters remained informative, particularly in patients with poor outcomes. However, because post-treatment measurements were obtained later in the ICU course, these values may reflect late-stage disease severity and ongoing immune dysregulation rather than treatment-associated effects. Therefore, pre- and post-treatment comparisons should be interpreted descriptively and should not be used to draw conclusions about the independent efficacy of hemadsorption.

Taken together, IG parameters should be regarded as readily available adjunctive indicators that complement established measures of disease severity, such as the SOFA score and lactate levels, but do not replace or serve as actionable biomarkers for therapeutic decision-making.

In addition to its strengths, the study has limitations that should be acknowledged. The single-center, retrospective design may limit the generalizability of the findings. Although standard sepsis management was applied, key treatment-related variables such as the exact timing of antibiotic administration, source control procedures, fluid resuscitation strategies, and vasopressor titration were not analyzed in detail, and variability in these components of care may have influenced immune trajectories (including IG dynamics) and clinical outcomes. The absence of a non-intervention control group also precludes a direct comparison between hemadsorption and standard therapy alone.

Post-treatment laboratory measurements were obtained at variable time points, limiting the ability to distinguish treatment-associated changes from time-dependent immune dynamics or late-course severity indicators. Moreover, detailed immunological and bone marrow–specific assessments (e.g., cytokine profiles of IL-6, TNF-α, and IL-10; endotoxin activity; ferritin; neutrophil functional assays; and markers of bone marrow activation) were unavailable, which limits mechanistic interpretation to descriptive and associative findings. Additional sensitivity or subgroup analyses based on post-treatment sampling time, baseline disease severity, or early mortality were not performed due to limited sample sizes within specific strata, which could increase the risk of underpowered comparisons and overinterpretation.

As a result, no inference can be made regarding the efficacy of hemadsorption or its direct immunological effects, and the present findings should be regarded as descriptive and hypothesis-generating. Future prospective, multicenter studies with predefined protocols and appropriate comparator groups are warranted, incorporating standardized sampling timepoints and integrated immunological profiling (including cytokines and endotoxin activity) to better clarify immune dynamics and clinical outcomes in sepsis.

In conclusion, this study describes inflammatory, immunological, and clinical changes observed during the ICU course of septic patients treated with hemadsorption. Alongside conventional inflammatory markers and organ dysfunction scores, IG parameters varied during the clinical course and may provide complementary information on immune dysregulation and disease severity in this patient group. Although no causal inferences can be made regarding the efficacy of hemadsorption, the observed findings should be interpreted within the context of comprehensive sepsis management. Accordingly, these results warrant confirmation in prospective, multicenter studies with appropriate control groups to refine patient selection and further elucidate immune and inflammatory dynamics during hemadsorption therapy in sepsis patients.

## Figures and Tables

**Table 1:** Baseline demographic and clinical characteristics of the patients.

Variable	Value
Female sex, n (%)	81 (41.3)
Male sex, n (%)	115 (58.7)
Age, years	57.0 ± 14.8
Charlson Comorbidity Index	2 (1–2)
APACHE II score	21.9 ± 3.4
Glasgow Coma Scale	12 (9–14)
Length of ICU stay, days	12 (8–17)
Mechanical ventilation, n (%)	162 (82.7)
Renal replacement therapy, n (%)	113 (57.7)
Mortality, n (%)	86 (43.9)

Data are presented as mean ± SD or median (interquartile range), as appropriate. Categorical variables are presented as n (%). ICU: Intensive Care Unit; APACHE II: Acute Physiology and Chronic Health Evaluation II.

**Table 2:** Comparison of clinical and laboratory parameters before and after hemadsorption.

Parameter	Pre-Treatment	Post-Treatment	*p*-Value
SOFA score	11 (8–12)	8 (3–11)	**<0.001**
Immature granulocytes, ×10^9^/L	0.45 (0.27–0.77)	0.09 (0.06–0.28)	**<0.001**
Immature granulocytes, %	2.50 (1.83–3.88)	0.80 (0.40–1.50)	**<0.001**
WBC, ×10^3^/µL	19,650 (15,520–25,910)	11,610 (8472.50–17,077.50)	**<0.001**
Neutrophils, ×10^3^/µL	16,840 (12,490–22,542.50)	9320 (6087–14,987.50)	**<0.001**
Lymphocytes, ×10^3^/µL	720 (475–957.50)	1100 (860–1502.50)	**<0.001**
Platelets, ×10^3^/µL	185,000 (133,000–242,250)	184,000 (136,500–223,750)	0.66
Monocytes, ×10^3^/µL	980 (680–1210)	560 (340–808.50)	**<0.001**
Lactate, mmol/L	11.15 (8.13–13.10)	2.10 (1.33–3.80)	**<0.001**
Albumin, g/dL	2.90 (2.60–3.40)	2.80 (2.50–3.10)	**<0.001**
CRP, mg/L	183 (135–219)	72.85 (29.78–111.00)	**<0.001**
D-dimer, mg/L	3.90 (1.54–9.45)	2.89 (1.68–6.49)	0.60
Procalcitonin, ng/mL	11.14 (3.60–26.53)	1.90 (0.71–4.50)	**<0.001**
Vasopressor dose, µg/kg/min	0.70 (0.40–1.00)	0.10 (0.03–0.50)	**<0.001**

Data are presented as median (interquartile range). Comparisons were performed using the Wilcoxon signed-rank test. SOFA: Sequential Organ Failure Assessment; WBC: White blood cell; CRP: C-reactive protein. Pre- and post-treatment refer to measurements before and after hemadsorption therapy. The statistically significant *p*-values are in bold.

**Table 3:** Comparison of clinical and laboratory parameters between survivors and non-survivors.

Variable	Non-Survivors	Survivors	*p*-Value
Female sex, n (%)	30 (37.0)	51 (63.0)	0.105
Male sex, n (%)	56 (48.7)	59 (51.3)	0.105
Age, years	58.1 ± 15.1	55.1 ± 13.3	0.38
Charlson Comorbidity Index	2.5 (2.0–3.0)	2.0 (1.0–2.0)	**<0.001**
Mechanical ventilation duration, days	8 (5–13)	7 (4.75–11)	0.215
Length of ICU stay, days	11 (7.75–16.25)	14.5 (9.0–20.5)	0.412
Glasgow Coma Scale	10 (8–12)	12 (8–14)	**<0.001**
SOFA score (pre-treatment)	12 (10.75–13.00)	8 (6–11)	**<0.001**
SOFA score (post-treatment)	10 (9–12)	3 (2.00–4.25)	**<0.001**
APACHE II score	28.0 ± 4.8	18.4 ± 5.9	**<0.001**
Immature granulocytes pre, ×10^9^/L	0.67 (0.45–0.89)	0.32 (0.21–0.44)	**<0.001**
Immature granulocytes post, ×10^9^/L	0.22 (0.10–0.38)	0.06 (0.03–0.07)	**<0.001**
Immature granulocytes %, pre	2.90 (2.30–4.13)	2.15 (1.28–3.10)	**<0.001**
Immature granulocytes %, post	1.40 (0.76–2.10)	0.50 (0.30–0.73)	**<0.001**
WBC pre, ×10^3^/µL	20,050 (15,557.5–26,252.5)	18,845 (15,197.5–25,027.5)	0.057
WBC post, ×10^3^/µL	14,435 (10,177.5–21,000)	9575 (8220–11,900)	**<0.001**
Neutrophils pre, ×10^3^/µL	17,220 (12,925–22,887.5)	16,295 (11,715–20,350)	**0.040**
Neutrophils post, ×10^3^/µL	12,725 (8495–17,502)	7060 (5280–9332.5)	**<0.001**
Lymphocytes pre, ×10^3^/µL	750 (547.5–1002.5)	640 (362.5–852.5)	**0.002**
Lymphocytes post, ×10^3^/µL	1035 (650–1432.5)	1245 (955–1542.5)	**0.009**
Platelets pre, ×10^3^/µL	158,000 (121,750–236,500)	208,000 (164,000–257,750)	**<0.001**
Platelets post, ×10^3^/µL	143,500 (101,000–189,000)	205,500 (185,500–255,250)	**<0.001**
Monocytes pre, ×10^3^/µL	965 (587.5–1195)	1020 (802.5–1250)	0.249
Monocytes post, ×10^3^/µL	605 (340–1000)	510 (340–730)	0.079
Lactate pre, mmol/L	11.65 (9.90–13.93)	9.25 (7.18–12.20)	**<0.001**
Lactate post, mmol/L	3.70 (2.10–5.03)	1.60 (1.10–2.04)	**<0.001**
Albumin pre, g/dL	2.90 (2.60–3.43)	2.90 (2.58–3.40)	0.427
Albumin post, g/dL	2.60 (2.40–2.90)	2.90 (2.70–3.20)	**<0.001**
D-dimer pre, mg/L	4.00 (2.00–10.00)	4.05 (1.40–11.98)	0.155
D-dimer post, mg/L	4.40 (2.52–9.70)	2.17 (1.45–3.73)	**<0.001**
CRP pre, mg/L	193.5 (135.5–245.0)	170.0 (135.0–203.25)	**0.025**
CRP post, mg/L	100.5 (65.0–145.0)	37.65 (21.25–70.50)	**<0.001**
Procalcitonin pre, ng/mL	12.10 (4.80–27.13)	10.70 (3.58–26.53)	0.823
Procalcitonin post, ng/mL	3.60 (1.38–9.09)	1.22 (0.39–2.08)	**<0.001**
Vasopressor dose pre, µg/kg/min	0.60 (0.40–1.00)	0.70 (0.40–0.93)	**0.007**
Vasopressor dose post, µg/kg/min	0.40 (0.10–0.90)	0.02 (0.01–0.05)	**<0.001**
Renal replacement therapy, n (%)	65 (75.6)	48 (43.6)	**<0.001**

Continuous variables are presented as mean ± SD or median (interquartile range), as appropriate. Categorical variables are presented as n (%). Comparisons were performed using the Mann–Whitney U test, Student’s t-test, or chi-square test, as appropriate. SOFA: Sequential Organ Failure Assessment; APACHE II: Acute Physiology and Chronic Health Evaluation II; WBC: White blood cell; CRP: C-reactive protein. Pre- and post-treatment refer to measurements before and after hemadsorption therapy. The statistically significant *p*-values are in bold.

**Table 4:** ROC Analyses of Immature Granulocyte Parameters in Relation to Mortality.

Variable	AUC (95% CI)	Cut-Off Value	Sensitivity	Specificity	*p*-Value
Immature granulocytes pre, ×10^9^/L	0.768 (0.699–0.836)	0.425	0.802	0.691	**<0.001**
Immature granulocytes post, ×10^9^/L	0.880 (0.826–0.935)	0.085	0.907	0.827	**<0.001**
Immature granulocytes %, pre	0.706 (0.633–0.780)	2.15	0.826	0.527	**<0.001**
Immature granulocytes %, post	0.812 (0.746–0.878)	0.85	0.709	0.864	**<0.001**

AUC: area under the curve; CI: confidence interval. Pre- and post-treatment refer to measurements before and after hemadsorption therapy. The statistically significant *p* values are in bold.

## Data Availability

The datasets used and analyzed during the current study are available from the corresponding author upon reasonable request.

## Supplementary Materials

The following supporting information can be downloaded at: https://www.mdpi.com/article/10.3390/jcm15031011/s1, Table S1: Univariate and multivariate logistic regression analyses for mortality; Figure S1. ROC Analyses of Immature Granulocyte Parameters in Relation to Mortality.

## Abbreviations

AUC	Area Under the Curve
APACHE II	Acute Physiology and Chronic Health Evaluation II
CBC	Complete Blood Count
CI	Confidence Interval
CRP	C-reactive protein
CRRT	Continuous Renal Replacement Therapy
CXCL12	C-X-C motif chemokine ligand 12
GCS	Glasgow Coma Scale
Hb	Hemoglobin
Hct	Hematocrit
ICU	Intensive Care Unit
IG	Immature Granulocyte
IG#	Immature Granulocyte Count
IG%	Immature Granulocyte Percentage
IL-6	Interleukin-6
IL-10	Interleukin-10
INR	International Normalized Ratio
LOS	Length of Stay
MV	Mechanical Ventilation
OR	Odds Ratio
PCT	Procalcitonin
ROC	Receiver Operating Characteristic
RRT	Renal Replacement Therapy
SOFA	Sequential Organ Failure Assessment
STROBE	Strengthening the Reporting of Observational Studies in Epidemiology
TNF-α	Tumor Necrosis Factor-alpha
WBC	White Blood Cell

## References

[1] Rudd K.E., Johnson S.C., Agesa K.M., Shackelford K.A., Tsoi D., Kievlan D.R., Colombara D.V., Ikuta K.S., Kissoon N., Finfer S. (2020). Global, Regional, and National Sepsis Incidence and Mortality, 1990–2017: Analysis for the Global Burden of Disease Study. Lancet.

[2] Gierlikowska B., Stachura A., Gierlikowski W., Demkow U. (2022). The Impact of Cytokines on Neutrophils’ Phagocytosis and NET Formation during Sepsis—A Review. Int. J. Mol. Sci..

[3] Dörge H., Schöndube F.A., Dörge P., Seipelt R., Voss M., Messmer B.J. (2003). Procalcitonin Is a Valuable Prognostic Marker in Cardiac Surgery but Not Specific for Infection. Thorac. Cardiovasc. Surg..

[4] Drifte G., Dunn-Siegrist I., Tissières P., Pugin J. (2013). Innate Immune Functions of Immature Neutrophils in Patients with Sepsis and Severe Systemic Inflammatory Response Syndrome. Crit. Care Med..

[5] Růžička K., Veitl M., Thalhammer-Scherrer R., Schwarzinger I. (2001). The New Hematology Analyzer Sysmex XE-2100: Performance Evaluation of a Novel White Blood Cell Differential Technology. Arch. Pathol. Lab. Med..

[6] Ha S.O., Park S.H., Park S.H., Park J.S., Huh J.W., Lim C.-M., Koh Y., Hong S.-B., Jang S. (2015). Fraction of Immature Granulocytes Reflects Severity but Not Mortality in Sepsis. Scand. J. Clin. Lab. Investig..

[7] Taneja R., Sharma A.P., Hallett M.B., Findlay G.P., Morris M.R. (2008). Immature Circulating Neutrophils in Sepsis Have Impaired Phagocytosis and Calcium Signaling. Shock.

[8] Bhansaly P., Mehta S., Sharma N., Gupta E., Gupta S. (2022). Evaluation of Immature Granulocyte Count as the Earliest Biomarker for Sepsis. Indian J. Crit. Care Med..

[9] van der Geest P.J., Mohseni M., Brouwer R., van der Hoven B., Steyerberg E.W., Groeneveld A.B. (2014). Immature Granulocytes Predict Microbial Infection in the ICU. J. Crit. Care.

[10] Lakshmipriya V., Kavitha K., Yogalakshmi E., Sridevi M. (2024). Clinical Utility of Automated Immature Granulocyte Measurement in Early Bacteremia Diagnosis. Cureus.

[11] Agnello L., Giglio R.V., Bivona G., Lo Sasso B., Ciaccio M. (2021). The Value of a Complete Blood Count for Sepsis Diagnosis and Prognosis. Diagnostics.

[12] Ayres L.S., Sgnaolin V., Munhoz T.P. (2019). Immature Granulocyte Index as an Early Marker of Sepsis. Int. J. Lab. Hematol..

[13] Evans L., Rhodes A., Alhazzani W., Antonelli M., Coopersmith C.M., French C., Machado F.R., Mcintyre L., Ostermann M., Prescott H.C. (2021). Surviving Sepsis Campaign: International Guidelines for Management of Sepsis and Septic Shock 2021. Intensive Care Med..

[14] Ferrari F., Clark W., Ronco C., Ronco C., Bellomo R., Kellum J.A., Ricci Z. (2018). Sorbents: From Basic Structure to Clinical Application. Critical Care Nephrology.

[15] Nierhaus A., Morales J., Wendt D., Scheier J., Gutzler D., Jarczak D., Born F., Hagl C., Deliargyris E., Mehta Y. (2022). Comparison of the CytoSorb® 300 mL and Jafron HA380 Hemoadsorption Devices: An In Vitro Study. Minim. Invasive Ther. Allied Technol..

[16] Bellomo R., Ankawi G., Bagshaw S.M., Baldwin I., Basu R., Bottari G., Cantaluppi V., Clark W., De Rosa S., Forni L.G. (2024). Hemoadsorption: Consensus Report of the 30th Acute Disease Quality Initiative Workgroup. Nephrol. Dial. Transplant..

[17] Gullotta G.S., De Feo D., Friebel E., Semerano A., Scotti G.M., Bergamaschi A., Butti E., Brambilla E., Genchi A., Capotondo A. (2023). Age-Induced Alterations of Granulopoiesis Generate Atypical Neutrophils that Aggravate Stroke Pathology. Nat. Immunol..

[18] Deniz M., Sahin Yildirim Z., Erdin Z., Alisik M., Erdin R., Yildirim M. (2025). Role of Immature Granulocytes in Monitoring Sepsis Treatment. BMC Anesthesiol..

[19] Rimmele T., Kellum J.A. (2012). High-Volume Hemofiltration in the Intensive Care Unit: A Blood Purification Therapy. Anesthesiology.

[20] Shum H.P., Chan K.C., Kwan M.C., Yan W.W. (2013). Application of Endotoxin and Cytokine Adsorption Haemofilter in Septic Acute Kidney Injury Due to Gram-Negative Bacterial Infection. Hong Kong Med. J..

[21] Cruz D.N., A Perazella M., Bellomo R., de Cal M., Polanco N., Corradi V., Lentini P., Nalesso F., Ueno T., Ranieri V.M. (2007). Effectiveness of Polymyxin B-Immobilized Fiber Column in Sepsis: A Systematic Review. Crit. Care.

[22] Cutuli S.L., De Rosa S., Ferrer R., Ruiz-Rodriguez J.C., Forfori F., Ronco C., Antonelli M., the EUPHAS2-G50 Collaborative Study Group (2023). Endotoxin Activity Trend and Multi-Organ Dysfunction in Septic Shock Patients Treated with Polymyxin-B Hemadsorption. Artif. Organs.

[23] Koc S., Celebi S., Hanikoglu F., Polat Y., Uysal B.B., Dokur M., Ozer T., Yavuzer S., Islamoglu M.S., Cengiz M. (2022). Can the Reduction of Cytokines Stop the Progression of Sepsis?. Cureus.

[24] Yakovlev A.Y., Ilyin Y.V., Bershadsky F.F., Selivanov D.D., Pevnev A.A., Trikole A.I., Popov A.Y., Pisarev V.M. (2025). Efficacy of Hemoadsorption in the Severe Course of COVID-19. Front. Med..

[25] Ryazanova D., Tobylbayeva Z., Mironova O., Kakenov E., Sazonov V. (2024). Comparison of CytoSorb and Jafron HA330 Hemoadsorption Devices in Pediatric Oncological Patients with Sepsis. J. Clin. Med..

[26] Paul R., Sathe P., Kumar S., Prasad S., Aleem M., Sakhalvalkar P. (2021). Clinical Outcomes with Extracorporeal Cytokine Adsorption in Sepsis and Septic Shock. World J. Crit. Care Med..

[27] Tanaka T., Fujino K., Tsujita Y., Matsumoto Y., Fujino M., Miyatake H., Mizumura N., Shimizu J., Kishimoto T., Shiomi N. (2025). Differential Impact of Polymyxin B Hemadsorption on Long-Term Mortality in Septic Shock. Artif. Organs.

[28] Jeon K., Lee N., Jeong S., Park M.J., Song W. (2021). Immature Granulocyte Percentage for Prediction of Sepsis in Severe Burn Patients. BMC Infect. Dis..

[29] Daix T., Jeannet R., Hernandez Padilla A.C., Vignon P., Feuillard J., François B. (2021). Immature Granulocytes in Pulmonary Bacterial Infections in Severe COVID-19. J. Intensive Care.

[30] Porizka M., Volny L., Kopecky P., Kunstyr J., Waldauf P., Balik M. (2019). Immature Granulocytes as a Sepsis Predictor in Cardiac Surgery. Interact. Cardiovasc. Thorac. Surg..

