# Supplementary material for: Immature Granulocyte Trajectories Following Hemadsorption as Indicators of Immune Dysregulation and Mortality
Source: J Clin Med. 2026 Jan 27;15(3):1011. doi: 10.3390/jcm15031011 (PMC12897782; doi:10.3390/jcm15031011)
Supplement: Supplementary file 1 [file jcm-15-01011-s001.zip › jcm-4089971-supplementary.pdf]

Supplementary Table S1. Univariate and multivariate logistic regression analyses for mortality

Variable	Univariate OR (95% CI)	p-value	Multivariate OR (95% CI)	p-value
Glasgow Coma Scale	0.88 (0.81–0.96)	0.005	—	—
SOFA score (pre-treatment)	1.89 (1.59–2.26)	<0.001	—	—
SOFA score (post-treatment)	4.83 (2.50–9.36)	<0.001	—	—
APACHE II score	1.43 (1.30–1.57)	<0.001	—	—
Immature granulocytes pre, $\times10^9$/L	38.66 (11.25–132.81)	<0.001	—	—
Immature granulocytes post, $\times10^9$/L	477.12 (44.62–5102.31)	<0.001	—	—
Immature granulocytes %, pre	1.69 (1.34–2.14)	<0.001	—	—
Immature granulocytes %, post	14.12 (6.17–32.31)	<0.001	14.21 (4.94–40.87)	**<0.001**
WBC pre, $\times10^3$/µL	1.00 (1.00–1.00)	0.096	—	—
WBC post, $\times10^3$/µL	1.00 (1.00–1.00)	<0.001	—	—
Neutrophils pre, $\times10^3$/µL	1.00 (1.00–1.00)	0.066	—	—
Neutrophils post, $\times10^3$/µL	1.00 (1.00–1.00)	<0.001	—	—
Lymphocytes pre, $\times10^3$/µL	1.001 (1.000–1.001)	0.038	—	—
Lymphocytes post, $\times10^3$/µL	0.999 (0.999–1.000)	0.029	—	—
Platelets pre, $\times10^3$/µL	1.00 (1.00–1.00)	0.030	—	—

Platelets post, ×10³/µL	1.00 (1.00–1.00)	<0.001	1.00 (1.00–1.00)	**<0.001**
D-dimer pre, mg/L	0.99 (0.96–1.02)	0.437	—	—
D-dimer post, mg/L	1.19 (1.10–1.29)	<0.001	1.12 (1.01–1.24)	**0.038**
Lactate pre, mmol/L	1.25 (1.14–1.37)	<0.001	—	—
Lactate post, mmol/L	4.11 (2.67–6.34)	<0.001	—	—
CRP pre, mg/L	1.01 (1.00–1.01)	0.024	0.99 (0.98–1.00)	0.082
CRP post, mg/L	1.02 (1.01–1.03)	<0.001	1.01 (1.00–1.01)	**0.011**
Procalcitonin pre, ng/mL	1.00 (0.99–1.01)	0.575	—	—
Procalcitonin post, ng/mL	1.14 (1.05–1.23)	0.001	—	—
Renal replacement therapy	4.00 (2.15–7.43)	<0.001	4.36 (1.38–13.78)	**0.012**

Odds ratios (ORs) are presented with 95% confidence intervals (CIs). Multivariate analysis was performed using backward stepwise logistic regression. Only variables with $p < 0.10$ in univariate analysis were included in the multivariate model.*SOFA: Sequential Organ Failure Assessment; APACHE II: Acute Physiology and Chronic Health Evaluation II; WBC: White blood cell; CRP: C-reactive protein. Pre- and post-treatment refer to measurements before and after haemadsorption therapy.*

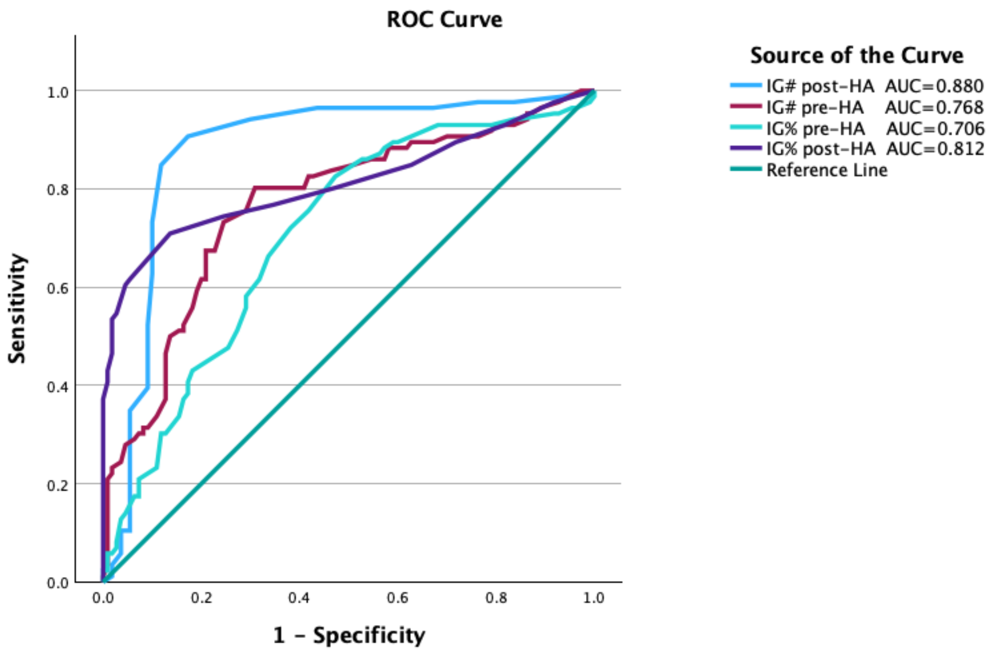

Figure S1. ROC Analyses of Immature Granulocyte Parameters in Relation to Mortality
